# Indoxyl Sulfate Induces Mesangial Cell Proliferation via the Induction of COX-2

**DOI:** 10.1155/2016/5802973

**Published:** 2016-10-23

**Authors:** Shuzhen Li, Sijie Cheng, Zhenzhen Sun, Harr-keshauve Mungun, Wei Gong, Jing Yu, Weiwei Xia, Yue Zhang, Songming Huang, Aihua Zhang, Zhanjun Jia

**Affiliations:** ^1^Department of Nephrology, Nanjing Children's Hospital, Nanjing Medical University, Nanjing, China; ^2^Institute of Pediatrics, Nanjing Medical University, Nanjing, China; ^3^Nanjing Key Laboratory of Pediatrics, Nanjing Children's Hospital, Nanjing Medical University, Nanjing, China

## Abstract

Indoxyl sulfate (IS) is one of important uremic toxins and is markedly accumulated in the circulation of end stage renal disease (ESRD) patients, which might contribute to the damage of residual nephrons and progressive loss of residual renal function (RRF). Thus this study was undertaken to investigate the role of IS in modulating mesangial cell (MC) proliferation and the underlying mechanism. The proliferation of MCs induced by IS was determined by cell number counting, DNA synthase rate, and cell cycle phase analysis. COX-2 expression was examined by Western blotting and qRT-PCR, and a specific COX-2 inhibitor NS398 was applied to define its role in IS-induced MC proliferation. Following IS treatment, MCs exhibited increased total cell number, DNA synthesis rate, and number of cells in S and G2 phases paralleled with the upregulation of cyclin A2 and cyclin D1. Next, we found an inducible inflammation-related enzyme COX-2 was remarkably enhanced by IS, and the inhibition of COX-2 by NS398 significantly blocked IS-induced MC proliferation in line with a blockade of PGE2 production. These findings indicated that IS could induce MC proliferation via a COX-2-mediated mechanism, providing new insights into the understanding and therapies of progressive loss of RRF in ESRD.

## 1. Introduction

Preservation of residual renal function (RRF) is important not only for predialysis ESRD patients but also for the patients undergoing the dialysis. RRF is a well-established predictor of the outcome and survival rate in dialysis patients [1]. Prospective randomized trials of dialysis adequacy and many observational studies have confirmed that the loss of RRF is highly associated with the mortality and morbidity in peritoneal dialysis (PD) patients [2, 3]. RRF in dialysis patients is pretty helpful in small-solute clearance, fluid balance, phosphorus control, and removal of middle-molecular uremic toxins, especially for the toxins relying on renal metabolism or tubular secretion, such as indoxyl sulfate (IS) [2, 4].

Evidence showed that serum concentration of IS was significantly elevated in ESRD patients [5]. IS is a protein-bound uremic toxin that derives from the metabolism of dietary tryptophan [6]. However, IS cannot be efficiently removed by conventional hemodialysis because of its high binding affinity to albumin in advanced chronic kidney disease (CKD) [7]. Thus, the urinary excretion of IS was considered to occur mainly by tubular secretion and glomerular filtration. The IS accumulated in serum accelerates tubular cell injury and induces subsequent interstitial fibrosis, thus acting as a nephrotoxin [5, 8–10]. Studies also indicated that IS could lead to complex redox alterations in mesangial cells (MCs) [11] and cell proliferation in vascular smooth muscle cells [12]. IS has been shown to have many pathological roles in uremia-related organ injuries. For example, it can increase the production of reactive oxygen species (ROS) and cause vascular wall remodeling and extracellular matrix deposition [13]. The MC proliferation and subsequent matrix synthesis could result in the glomerular impairment and RRF loss. However, the role of IS in mediating MC proliferation still needs evidence.

COX-2, an inducible isoform of COXs, is expressed in the macula densa of the juxtaglomerular apparatus, cortical thick ascending limb of Henle (cTAL) in the renal cortex, and interstitial cells in the renal medulla [14]. PGE2 as one of five major prostaglandins is synthesized by COX-2-related enzyme cascade and regulates glomerular filtration, renin release in the renal cortex, and tubular absorption of sodium and/or water in the medulla [15]. Accumulating evidence indicated that COX-2 contributes to a number of inflammatory diseases [16, 17] possibly via PGE2-mediated mechanisms. Recently, a report demonstrated that COX-2 was inducible in the MCs in response to sphingosine 1-phosphate stimulation [18]. Thus, in the present study, we fully studied the roles of IS in MCs proliferation and COX-2 regulation, as well as the role of COX-2 in the proliferative process of MCs challenged by IS.

## 2. Materials and Methods

### 2.1. Materials

IS was purchased from Sigma (St. Louis, MO). Dulbecco's modified Eagle's medium (DMEM), fetal bovine serum (FBS), penicillin-streptomycin, and trypsin-EDTA solution were purchased from Gibco (Invitrogen, Grand Island, NY). Cyclin D1 mouse monoclonal antibody and cyclin A2 rabbit polyclonal antibody were from Abcam. COX-2 mouse monoclonal antibody was purchased from Cayman Chemicals (Ann Arbor, MI). Anti-GAPDH (ab9485) was provided by Cell Signaling Technology (Danvers, MA). The PGE2 enzyme immunoassay kit was from Cayman Chemicals (Ann Arbor, MI). COX-2 inhibitor, NS-398, was bought from Beyotime (Shanghai, China).

### 2.2. MCs Culture

The mouse MC line HBZY-1 was obtained from the China Center for Type Culture Collection (CCTCC Wuhan, China). Cells were maintained at 37°C in a humidified 5% CO_2_ atmosphere in DMEM which contained 5.6 mM glucose, 10% fetal bovine serum (FBS; GIBCO), 100 U/mL penicillin, 100 mg/mL streptomycin, 44 mM NaHCO_3_, and 14 mM 4-(2-hydroxy-ethyl)-1-piperazineethanesulfonic acid. After MCs were cultivated to 60%–70% confluence, they were treated with IS for 24 h at different doses (0, 250, and 500 *μ*M) with or without COX-2 inhibitor NS-398 treatment at a dose of 10 *μ*M.

### 2.3. Cell Cycle Analysis

MCs were treated with vehicle and the indicated doses of IS with or without COX-2 inhibitor in serum-free DMEM for 24 h. Cells were washed twice with PBS before digestion with 0.25% trypsin and fixed in 70% ethanol for at least 2 h at 4°C. Then cells were collected by centrifugation, treated with RNase, and stained with propidium iodide by using cell cycle detection kit (KeyGEN, Shanghai, China). The number of cells in G1, S, and G2/M cell cycle phases was analyzed by flow cytometry (BD FACS Calibur flow cytometer, Bedford, MA), and data analysis was performed with modifit 3.0 software.

### 2.4. Quantitative Real-Time PCR

Total RNA was from cultured MCs by using a TRIzol reagent (TaKaRa) according to the manufacturer's protocol. Reverse transcription was performed using a PrimeScript RT reagent Kit (TaKaRa) according to the manufacturer's protocol. Oligonucleotides (cyclin D1: forward, 5′-CGC CCT CCG TTT CTT ACT TC-3′, and reverse, 5′-GCA GTC AGG GGA ATG GTC T-3′; cyclin A2: forward, 5′-AAG ATG CCC TGG CTT TTA GTG-3′, and reverse, 5′-TAACATTCACTGGCTTTTCGTCT-3′; Cyclooxygenase-2: forward, 5′-AGGACTCTGCTCACGAAGGA-3′, and reverse, 5′-TGACATGGATTGGAACAGCA-3′; and GAPDH: forward, 5′-GTCTTCACTACCATGGAGAAGG-3′, and reverse, 5′-TCATGGATGACCTTGGCCAG-3′) were designed using Primer 5 software (available at http://frodo.wi.mit.edu/) and synthesized by Invitrogen. Real-time PCR amplification was performed using the ABI 7500 Real-Time PCR Detection System (Foster City, CA) by using SYBR Premix Ex Taq (TaKaRa). The cycling program consisted of a preliminary denaturation (95°C for 10 min), followed by 40 cycles (95°C for 15 s and 60°C for 1 min). Relative gene expression of mRNA was normalized to GAPDH and calculated using the ΔΔCt method.

### 2.5. Western Blotting Analysis

At the indicated time points, MCs were rapidly washed with ice-cold PBS and lysed on ice in lysis buffer containing protease inhibitors. After centrifugation, the protein level was determined using a micro BCA protein assay kit with bovine serum albumin as a standard (Pierce, Thermo). Sixty micrograms of cellular proteins were separated by SDS-PAGE and transferred onto PVDF membranes (Bio-Rad). The membranes were blocked by TBS-T (0.1% Tween 20 in TBS) containing 5% nonfat milk for 1 h at room temperature, and then incubated with primary antibodies directed against cyclin D1 (1 : 1000), cyclin A2 (1 : 500), and COX-2 (1 : 500) by overnight incubation at 4°C, followed by the addition of HRP-labeled secondary antibodies at room temperature for 1 h. GAPDH was used as an internal standard control. Band intensity was measured using Image J software (NIH, Bethesda, MD, USA).

### 2.6. Enzyme Immunoassay

Cell culture medium was centrifuged for 5 min at 12,000 ×g. The concentration of PGE2 in the medium was determined by enzyme immunoassay (Cayman Chemical), according to the manufacturers' instructions.

### 2.7. Data Analysis

Data are presented as means ± SE. Statistical analysis was performed using ANOVA analysis followed by a Bonferroni posttest. *P* < 0.05 was considered statistically significant.

## 3. Results

### 3.1. IS-Induced MCs Proliferation

**Figure 1:**
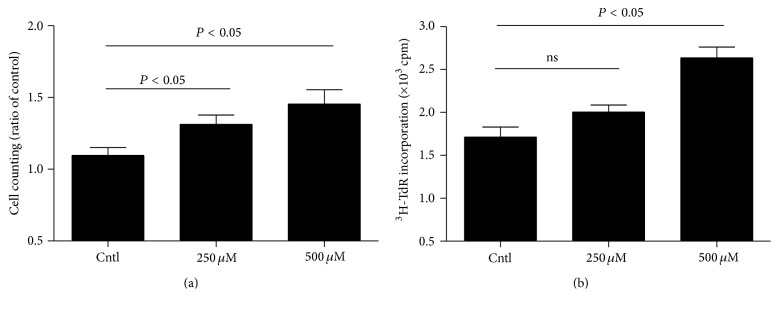
Effect of indoxyl sulfate (IS) on cell proliferation in MCs. After mesangial cells were cultivated to 60%–70% confluence, they were treated with IS for 24 h at different doses (0, 250, and 500 *μ*M) and cell proliferation was measured by cell counting (a) and [^3^H] thymidine (^3^H-TdR) incorporation (b). Values are means ± SD; *n* = 6 in each group.

To investigate whether IS could induce MC proliferation, we treated the MCs with IS at the doses of 250 *μ*M and 500 *μ*M, respectively. Cell proliferation was firstly examined by direct cell counting under the microscope and DNA synthesis rate ([^3^H] thymidine uptake). As shown by the data, IS treatment for 24 h at the doses of 250 and 500 *μ*M moderately but significantly increased the total cell number by 21% and 35%, respectively (Figure 1(a)). To further confirm this result, we examined DNA synthesis rate. Similarly, the amount of [^3^H] thymidine uptake in IS-treated cells was also increased in a dose-dependent manner (Figure 1(b)). The data suggested that IS played a role in promoting mesangial cell proliferation.

### 3.2. IS-Induced Cell Cycle Progression in MCs

**Figure 2:**
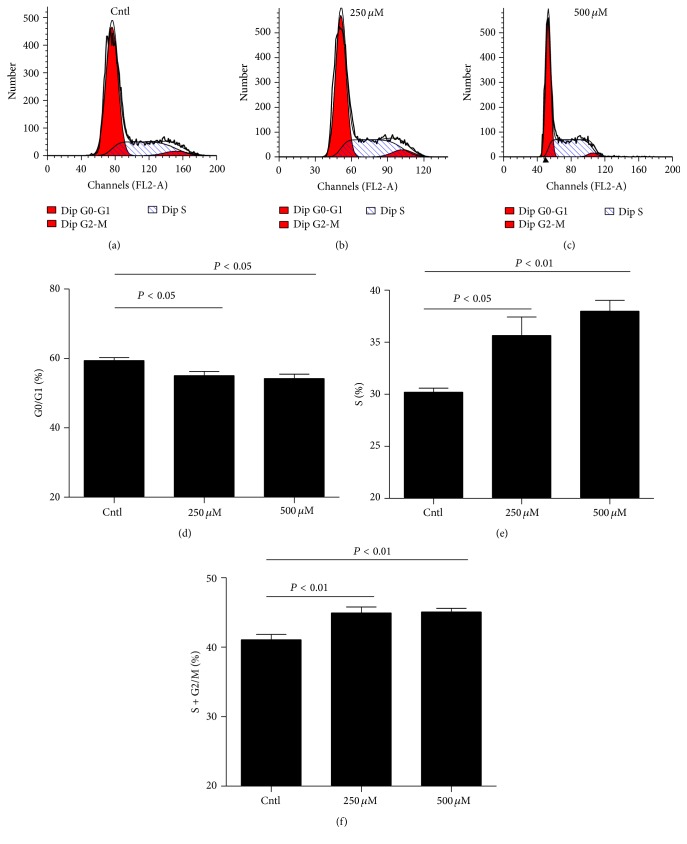
Effect of IS on cell cycle progression in MCs. The percentage of cells at different cell cycle phases was detected by flow cytometry after MCs were treated with the indicated doses of IS for 24 h. (a–c) Representative images of cell cycle with different doses of IS. (d–f) Percentage of cells at S, G1/G0, and (S + G2)/M phases. Values are means ± SD; *n* = 6 in each group.

In order to further validate the conclusion shown above, we measured the cell cycle by flow cytometry in MCs exposed to different doses of IS. As shown by data, IS caused a moderate but significant decrease of MC numbers in the G1/G0 phase and increase of cell numbers in the S phase (Figures 2(a)–2(f)). Cell cycle analysis revealed that IS can stimulate cell cycle progression in MCs.

### 3.3. IS Upregulated Cyclin D1 and Cyclin A2 in MCs

**Figure 3:**
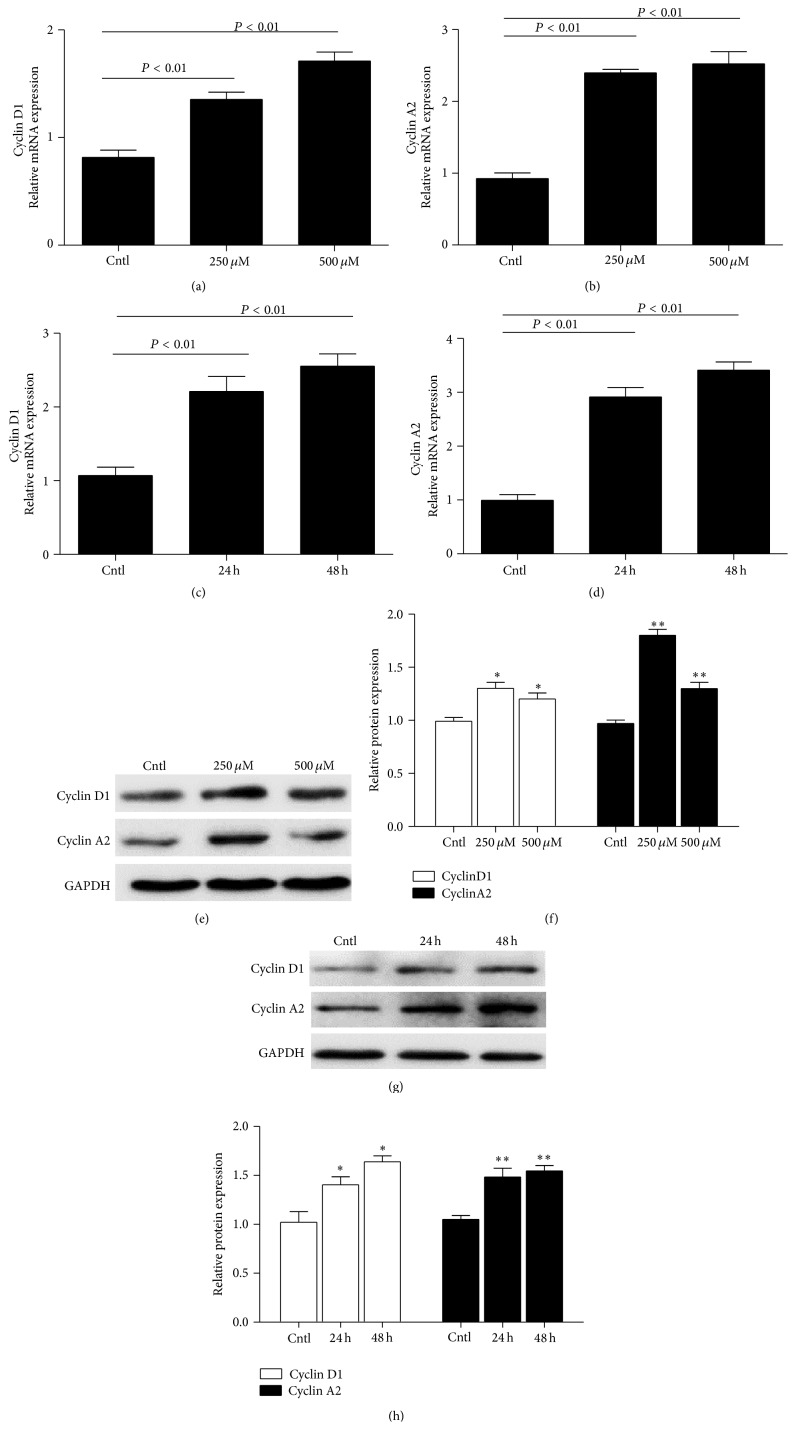
Effects of IS on the expressions of cyclin D1 and cyclin A2 in MCs. After MCs were treated with IS, cyclin D1 (a) and cyclin A2 (b) mRNA levels were elevated in a dose-dependent manner following 24 h treatment. Meanwhile, a time-dependent induction of cyclin D1 and cyclin A2 mRNA expressions was tested (c and d). Protein levels of cyclin D1 and cyclin A2 were also detected using dose- and time-dependent experiments (e–h). All values are means ± SD; *n* = 6 in each group; ^*∗*^ *P* < 0.05 versus control and ^*∗∗*^ *P* < 0.01 versus control.

To further investigate the IS effect on MC proliferation, we measured the expressions of some key cell cycle-related proteins. Here we found that IS strikingly increased the mRNA levels of cyclin D1 and cyclin A2 in dose- and time-dependent manners determined by qRT-PCR (Figures 3(a)–3(d)). By Western blotting, we observed a similar pattern of the protein expressions of cyclin D1 and cyclin A2 as their mRNA regulation (Figures 3(e)–3(h)).

### 3.4. IS Upregulated COX-2 Expression in MCs

**Figure 4:**
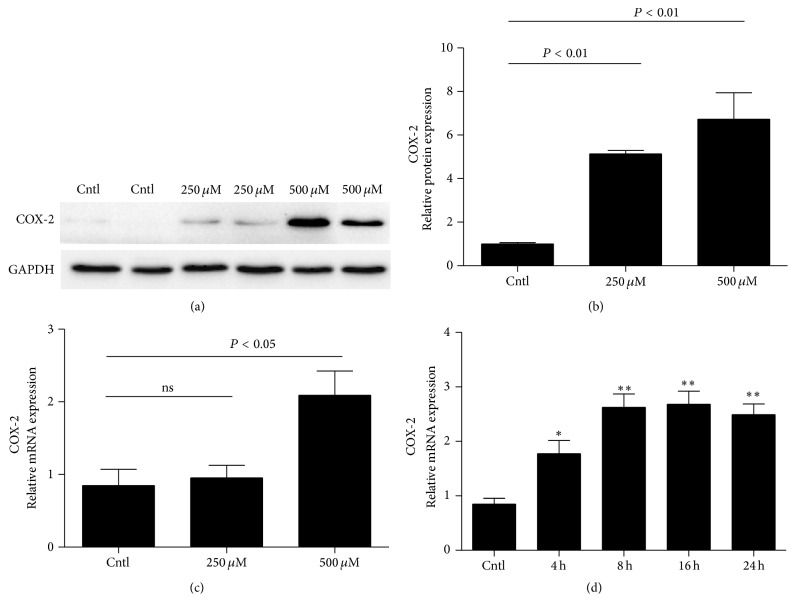
Expression of COX-2 in IS-induced mouse MCs. MCs were treated with the indicated doses of IS (0, 250, and 500 *μ*M) for 24 h and then COX-2 protein (a and b) and mRNA (c) and protein expressions were analyzed by Western blotting and qRT-PCR. A time course analysis of COX-2 mRNA expression following IS treatment at a dose of 500 *μ*M was also examined (d). Values are means ± SD; *n* = 6 in each group; ^*∗*^ *P* < 0.05 versus control and ^*∗∗*^ *P* < 0.01 versus control.

To study the possible involvement of COX-2 in IS-induced MS proliferation, we measured COX-2 expression by Western blotting and qRT-PCR. As shown by data, COX-2 protein was dose-dependently elevated (Figures 4(a) and 4(b)). For the mRNA expression, 500 *μ*M but not 250 *μ*M increased COX-2 mRNA level (Figure 4(c)). Using 500 *μ*M IS, we found a time-dependent induction of COX-2 mRNA expression (Figure 4(d)). These results indicated that COX-2 could be induced by IS in MCs.

### 3.5. Silencing COX-2 Blocked IS-Induced Cell Cycle Progression in MCs

**Figure 5:**
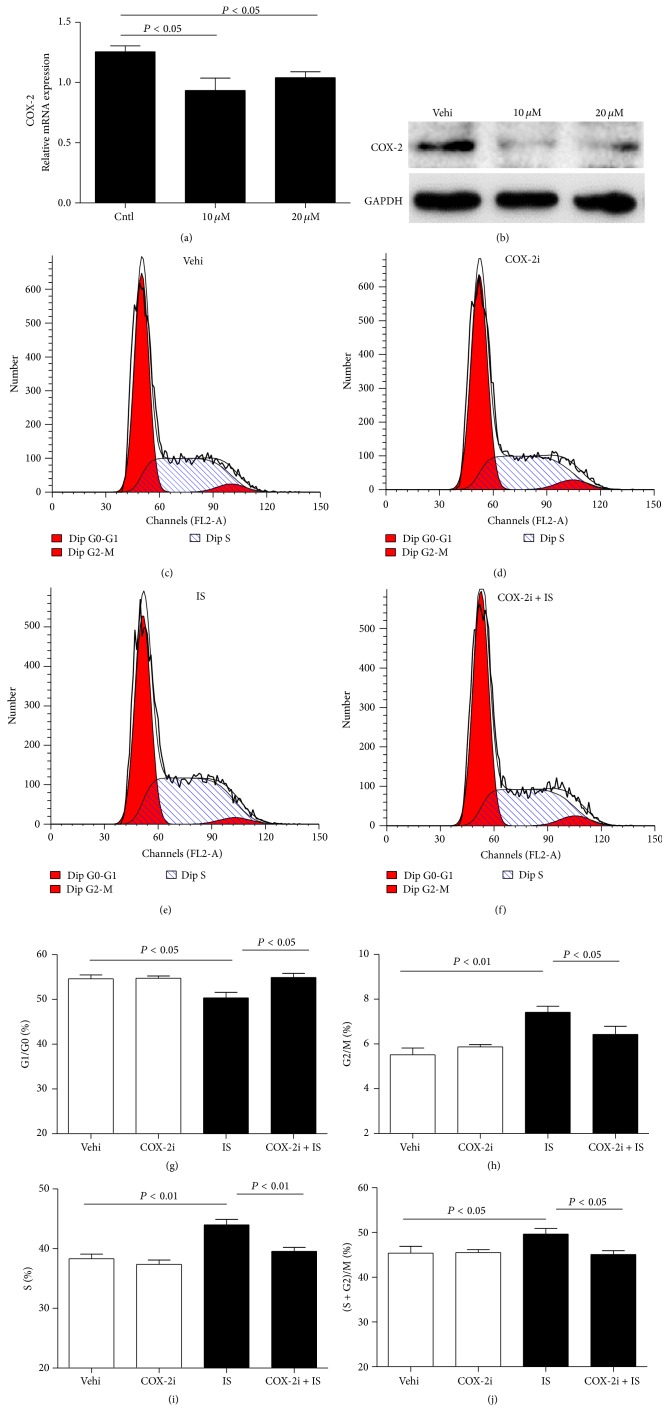
Effects of COX-2 specific inhibitor NS398 on cell cycle progression after IS treatment. The cells were treated with NS398 (10 *μ*M) for 12 h before IS (500 *μ*M) administration. (a) qRT-PCR analysis of COX-2 after COX-2 inhibitor (NS398, 10 *μ*M, and 20 *μ*M) treatment in MCs. (b) Western blotting analysis of COX-2 expression after COX-2 inhibitor (10 *μ*M and 20 *μ*M) treatment in MCs. (c–f) Representative images of cell cycle following the treatments of IS and/or COX-2 inhibitor. (g–j) Percentage of cells at G1/G0 (g), G2/M (h), S (i), and (S + G2)/M (j) phases following the treatments of IS and/or COX-2 inhibitor. Values are means ± SD; *n* = 6 in each group.

**Figure 6:**
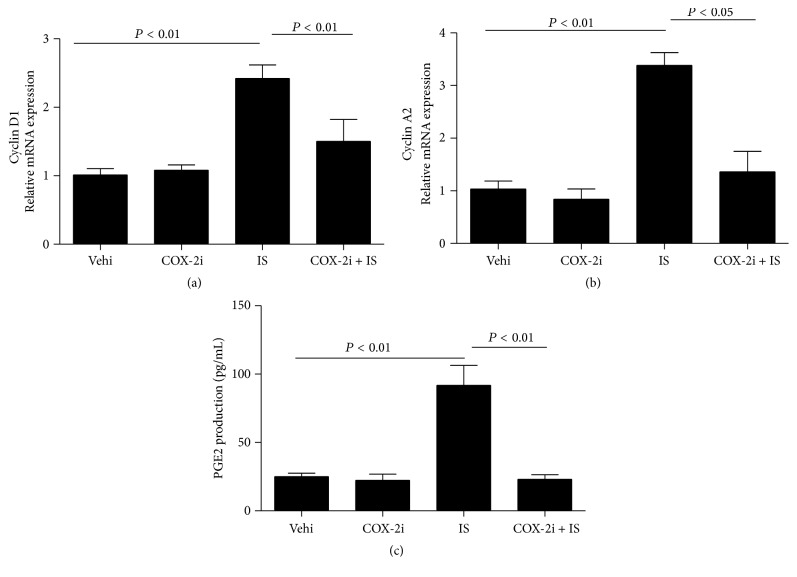
Effects of COX-2 inhibition on the mRNA expressions of cyclin D1 and cyclin A2 and the production of PGE2. (a) mRNA expression of cyclin D1 determined by qRT-PCR; (b) mRNA expression of cyclin A2 determined by qRT-PCR; (c) enzyme immunoassay of PGE2 in the medium. Values are means ± SD; *n* = 6 in each group.

To examine the role of COX-2 in IS-induced proliferation in MCs, specific COX-2 inhibitor was applied to MCs. As shown in Figures 5(a) and 5(b), COX-2 inhibitor at 10 and 20 *μ*M lowered COX-2 expression. Further, we observed that COX-2 inhibition decreased the cell number in S phase and increased cell number in G1/G0 phase (Figures 5(c)–5(j)). Moreover, we also found that COX-2 inhibitor markedly attenuated IS-induced cyclin D1 and cyclin A2 expression at mRNA levels (Figures 6(a) and 6(b)). These data highly suggested COX-2 played an important role in MC proliferation.

### 3.6. Silencing COX-2 Significantly Blocked IS-Induced PGE2 Production

To further examine the efficiency of COX-2 inhibitor in the study, we measured PGE2 production in the medium. As shown by Figure 6(c), IS treatment increased PGE2 level by 3.8-fold, which was entirely abolished by COX-2 inhibition.

## 4. Discussion

RRF is very important for both predialysis and dialysis patients. Studies have demonstrated that the loss of RRF is a powerful predictor of mortality and morbidity in peritoneal dialysis (PD) patients [1–3]. RRF is pretty helpful for the removal of middle and larger molecular weight toxins [2, 4]. IS, as one of the most known uremic toxins, is markedly accumulated in the serum of dialysis patients and accelerates the progression of disease [6, 19]. In this study, we found that IS could moderately but significantly stimulate MC proliferation via a COX-2-mediated mechanism, which might contribute to the progressive loss of RRF.

Cell proliferation is ultimately regulated at cell cycle process including four stages of G1, S, G2, and M with important checkpoints in G1 and G2. Cyclin D1 controls cell cycle progression through the G1 phase and G1-to-S transition [20], and cyclin A-associated kinase activity is required for the entry into S, completion of S, and entry into M phase [21]. Thus the expressions of cyclin D1 and cyclin A2 were chosen as markers for cell cycle progression. Dose-response experiments showed that IS significantly induced cyclin A2 and cyclin D1 expression in MCs consistent with the results which were demonstrated in rat MCs [22]. Similarly, the increase of cell numbers in the S phase was also observed following IS treatment. These findings highly suggested that IS served as a contributor of cell cycle progression and cell proliferation in MCs.

Next, we examined the possible mechanism mediating IS effect on MC proliferation. By reviewing the literature, recent evidence indicated that an inducible inflammatory enzyme COX-2 can be induced in MCs in response to sphingosine 1-phosphate stimulation [18]. Following this notion, we examined the regulation of COX-2 in MCs following IS treatment. Interestingly, IS remarkably elevated COX-2 expression at both mRNA and protein levels in dose- and time- dependent manners. Meantime, IS also elevated PGE2 production. These data indicated that IS could directly stimulate COX-2 upregulation and PGE2 induction in MCs. In order to further define the role of COX-2 in this cell cycle progression, COX-2 specific inhibitor was applied to the cells before IS administration. As expected, COX-2 inhibitor blunted the COX-2 expression. Meanwhile, reduction of cell cycle-related proteins of cyclin D1 and A2 was also significantly attenuated by COX-2 inhibition in line with a blockade of PGE2 induction.

All these data indicated a significant role of IS in promoting MC proliferation via activating COX-2. The proliferation of MCs has to be attributable to the progressive impairment of residual nephrons, as well as the loss of RRF in ESRD patients to some extent. Considering the important role of RRF in maintaining a better homeostasis of internal environment and better life quality of patients, targeting IS and COX-2 might be useful for the preservation of RRF.
